# Shared Decision-Making in Dialysis Modality Selection Among Adults with Advanced Kidney Disease in Saudi Arabia: A Cross-Sectional Study

**DOI:** 10.3390/healthcare14162610

**Published:** 2026-08-19

**Authors:** Aljawharah M. Almari, Homood A. Alharbi, Ann Bonner, Jeanette Finderup, Hayfa Almutary

**Affiliations:** 1 College of Nursing, King Saud University, Riyadh 12372, Saudi Arabia or amalmaree@pnu.edu.sa (A.M.A.); homalharbi@ksu.edu.sa (H.A.A.); 2 College of Nursing, Princess Nourah Bint Abdulrahman University, Riyadh 84428, Saudi Arabia; 3 School of Nursing and Midwifery, Griffith University, Brisbane 4000, Australia; a.bonner@griffith.edu.au; 4 Department of Renal Medicine, Aarhus University Hospital, 8200 Aarhus, Denmark; jeanette.finderup@rm.dk; 5 Department of Clinical Medicine, Aarhus University, 8200 Aarhus, Denmark; 6 Medical-Surgical Nursing Department, Faculty of Nursing, King Abdulaziz University, Jeddah 22246, Saudi Arabia

**Keywords:** advanced chronic kidney disease (CKD), dialysis, dialysis modality, nursing, shared decision-making

## Abstract

**Highlights:**

**What are the main findings?**

- Participants reported a mean standardized SDM-Q-9 score of 64.7%.
- Collaborative deliberation and shared treatment selection were less consistent.

**What are the implications of the main findings?**

- The findings highlight opportunities to enhance shared decision-making processes within multidisciplinary kidney-care teams.
- Existing evidence indicates that multidisciplinary strategies, such as communication training, decision coaching, and patient decision aids, can facilitate patient involvement in treatment decision-making.

**Abstract:**

**Aim:** Shared decision-making is increasingly recognized as a cornerstone of person-centered care. The aim of this study to assess the level of adults’ involvement in shared decision-making regarding dialysis modality choice and to identify its factors. **Design:** A cross-sectional correlational design was used. **Methods:** Data were collected from adults with advanced chronic kidney disease (CKD) at two Saudi hospitals between July and December 2024. Shared decision-making was measured using the Arabic version of the 9-item Shared Decision-Making Questionnaire. Perceived health status was assessed using a single item from the Kidney Disease Quality of Life questionnaire. Education and support were assessed using two dichotomous items. Data were analyzed using descriptive statistics, *t*-tests, ANOVA, and multiple regression analysis. **Results:** A total of 294 participants were included. The mean shared decision-making score was 64.7%. The highest mean item scores were observed for clarifying that a decision was required (4.65 ± 1.861) and assisting patients in understanding the available information (4.60 ± 1.682). A multiple regression analysis examined the contributions of attendance at a kidney treatment options education class and perception of health to shared decision-making perception among participants. The final model was statistically significant, *F*(2, 291) = 16.60, *p* < 0.001, and accounted for 10.2% of the variance in shared decision-making perception (*R*^2^ = 0.102, adjusted *R*^2^ = 0.096). Attendance at a kidney treatment options education class was a significant positive predictor in both steps (*B* = 0.436, *SE* = 0.086, *β* = 0.282, *p* < 0.001), and perception of health contributed uniquely in the final model (*B* = 0.056, *SE* = 0.025, *β* = 0.127, *p* = 0.024). Education-class attendance exerted a stronger unique effect than perception of health. Together, these two factors explained a modest but statistically significant portion of the variance in shared decision-making perception. **Conclusions:** Adults with advanced CKD in Saudi Arabia reported moderate-to-high levels of perceived shared decision-making, although collaborative deliberation and joint treatment selection remained limited. Attendance at a kidney treatment options education class showed the strongest unique association with higher shared decision-making perception, followed by perceived health status. Although these factors together explained only a modest proportion of the variance, the findings underscore the value of structured education and support in promoting patient involvement in treatment decisions. Further research should evaluate whether integrating structured kidney treatment options education into pre-dialysis care pathways improves person-centered care and patient engagement in modality selection.

## 1. Introduction

Chronic kidney disease (CKD) is an irreversible, progressive condition associated with high morbidity, mortality, and significant psychosocial burden [1,2]. When individuals reach advanced CKD, initiation of kidney replacement therapy (KRT) becomes unavoidable, with hemodialysis representing the most common treatment approach. Accordingly, involving adults in decision-making regarding the treatment pathways is a critical component of pre-dialysis care to ensure that treatment outcomes align with their values and preferences. A more suitable conceptual framing for this study is the shared decision-making (SDM) process that occurs throughout dialysis treatment planning. Instead of viewing dialysis modality selection solely as a sequence of treatment choices, SDM frames decision-making as a collaborative process in which healthcare professionals and adults jointly recognize the existence of a decision, exchange evidence-based information about available treatment options, explore the individual’s values, preferences, and goals, deliberate on the advantages and disadvantages of each option, and reach a mutually agreed treatment decision [3]. In the context of advanced CKD, this collaborative process may involve decisions regarding conservative kidney management, KRT, dialysis modality, and treatment setting, with decisions revisited as clinical circumstances and patient preferences change.

In this study, SDM is conceptualized as a set of observable clinical behaviors during healthcare consultations. These behaviors include identifying the need for a decision, presenting treatment options, supporting patient understanding, eliciting preferences, deliberating collaboratively, and reaching a shared decision. The 9-item Shared Decision-Making Questionnaire (SDM-Q-9) is used to assess participants’ perceptions of SDM in the present study [4,5].

SDM emphasizes a collaborative approach in which adults and healthcare professionals make shared decisions about the best treatment plan and appropriate options [6,7]. The literature highlights that this collaboration enhances adults’ self-management capabilities [3,8,9,10].

In kidney care, multidisciplinary kidney-care teams play a central role in facilitating informed decision-making through patient education, values clarification, and ongoing support across the CKD trajectory [11,12]. Their continuous contact with patients and families places multidisciplinary kidney-care teams in a unique position to support communication, patient understanding, and values-based decision-making. Decision coaching has been shown to reduce decisional uncertainty, improve understanding of treatment options, support alignment of decisions with individual values, and strengthen person-centered care approaches [6,13,14]. This may facilitate a smoother transition to either conservative kidney management or KRT options and reflects the quality of multidisciplinary kidney-care teams. Understanding how adults with advanced CKD experience involvement in dialysis decision-making is important for developing interventions that support person-centered kidney care, including multidisciplinary approaches described in the literature.

## 2. Background

The literature indicates that unplanned initiation of dialysis and limited involvement in treatment planning are associated with increased morbidity and mortality, higher healthcare costs, and dissatisfaction with care [15,16,17]. Consequently, strengthening pre-dialysis care requires approaches that actively support SDM. Evidence from studies involving adults with advanced CKD indicates that interventions such as decision coaching, patient decision aids, and communication training for healthcare professionals can enhance adults’ participation in treatment decisions and improve the quality of dialysis modality choices [3,12,13,18]. These approaches move beyond information provision alone by supporting adults in comparing treatment options, exploring their values and preferences, and participating meaningfully in decisions about KRT or conservative kidney management.

SDM refers to a collaborative process in which healthcare professionals and adults work together to make health decisions by considering the best available evidence alongside the individual’s values, preferences, and circumstances [4]. Within nursing practice, SDM is closely linked to relational and person-centered approaches that emphasize dialog, trust, and support for patient autonomy [19]. Contemporary conceptualization emphasizes deliberation and partnership, in which clinicians support individuals in understanding available options and in considering what matters most to them when choosing among treatments [12,20,21]. Evidence indicates that SDM is associated with improved patient satisfaction, increased adherence, better psychological outcomes, improved self-management, and reduced healthcare resource utilization [3,12,22]. However, studies indicate that age, education, and income may influence SDM among adults undergoing dialysis treatment [18,23,24].

Adults’ involvement in dialysis decision-making has been advocated globally for more than a decade, yet implementation in nephrology settings remains challenging [22]. Barriers include time constraints, cultural influences, limited use of patient decision aids, and a lack of skills in decision coaching, SDM, and communication [3,11,25]. Additional barriers include patient–clinician trust, physicians’ attitudes, misaligned incentives, and structural constraints [26,27]. Overcoming these obstacles requires not only clinical knowledge but also nursing competencies in decision coaching, SDM, and communication skills. The SDM process involves deliberation and consultation to empower patients to understand available treatment options and select the most suitable modality. Multidisciplinary kidney-care teams are well positioned to support patients in comparing options and clarifying their values and preferences related to the decision, which empowers patients to participate meaningfully in dialysis decision-making.

Previous studies have shown that many adults with advanced CKD report limited involvement in treatment decisions and insufficient discussion of available options, including hemodialysis, peritoneal dialysis, and conservative management [3,12,18]. Evidence also suggests that the quality of SDM varies across healthcare systems and cultural contexts. However, most of these studies were conducted in Western countries. A growing body of literature indicates that the degree of patient involvement in dialysis decisions varies widely and is influenced by factors such as clinical stage, educational attainment, and access to structured decision-support interventions [3,18,22]. Dialysis modality selection is determined by a diverse range of factors beyond SDM. Adults consider anticipated quality of life, lifestyle compatibility, perceived treatment burden, self-management capacity, family and social support, employment, travel requirements, and concerns about complications when choosing among hemodialysis, peritoneal dialysis, conservative kidney management, or transplantation. Clinical factors, such as infection risk, comorbidity burden, functional status, and home suitability, further inform treatment recommendations and patient preferences. Therefore, dialysis modality selection is a multifaceted process in which SDM serves as a framework for integrating clinical evidence with individual values, preferences, and life circumstances, rather than being the sole determinant of treatment choice [3,12,15,20].

Researchers in Saudi Arabia have mainly explored perceptions, awareness, and preferences regarding SDM among the general population and reported positive attitudes toward patient participation in clinical decisions [28,29,30]. For instance, Amin et al. [31] examined the role of SDM in promoting prostate cancer screening among the Saudi community and highlighted the importance of activating the role of primary healthcare physicians in the SDM process. In another study, the authors examined the factors influencing SDM awareness among hemodialysis nurses and reported that professionalism, empathy, and clinical decision-making ability influenced SDM awareness [32]. Previous studies indicate that many adults with advanced CKD report limited involvement in dialysis treatment decisions, and that participation in SDM is shaped by clinical, educational, and healthcare system factors. However, most existing evidence originates from Western countries, where healthcare systems, care models, and cultural expectations regarding patient participation differ markedly from those in Saudi Arabia. In the Saudi context, research has primarily focused on awareness of SDM or healthcare professionals’ perspectives, rather than on patients’ actual involvement in dialysis modality decisions and the associated factors. To date, there is limited evidence regarding how adults with advanced CKD experience SDM during dialysis modality selection in Saudi nephrology settings, as well as the patient-related or clinical factors associated with greater involvement. The present study addresses this gap by providing quantitative evidence on patients’ perceived involvement in SDM for dialysis modality selection and identifying factors associated with this involvement among adults with advanced CKD in Saudi Arabia.

## 3. Methods

### 3.1. Aims

The aim of this study was to assess the level of involvement in SDM regarding dialysis modality and to identify its predictors. Due to the limited evidence available in Saudi Arabia, this study was exploratory in nature, and therefore, a pre-specified hypothesis was not tested.

### 3.2. Design

This study used a cross-sectional analytical design. It was conducted in accordance with the Strengthening the Reporting of Observational Studies in Epidemiology (STROBE) guidelines for cross-sectional research (see Supplementary File S1).

### 3.3. Setting, Participants, and Data Collection

Data were collected from two large government hospitals in Riyadh, Saudi Arabia, between July 2024 and December 2024. Participants were recruited from the hemodialysis unit, peritoneal dialysis clinic, and outpatient nephrology clinic. Inclusion criteria were patients (1) aged above 18 years, (2) diagnosed with advanced CKD, (3) hemodynamically stable, and (4) able to communicate effectively in Arabic. Participants were excluded if they had documented cognitive impairment; participants with CKD grades not meeting criteria for advanced CKD were also excluded. Sample size was initially estimated using the Raosoft Sample Size Calculator (Raosoft, Inc., Seattle, WA, USA), assuming a population of 514 adults with advanced CKD, a 95% confidence level, and a 5% margin of error. This yielded a minimum required sample of 223 participants. In addition, because the primary analysis involved multivariable linear regression, sample adequacy was evaluated using the recommendation of approximately 20 participants per candidate predictor variable to ensure stable regression estimates. With 13 candidate predictors, a minimum sample of 260 participants was required. The final sample comprised 294 participants, exceeding both the population-based and regression-based requirements, providing approximately 23 participants per predictor variable, and reducing the risk of model overfitting [33].

After ethical approval, the first author collaborated with head nurses to identify eligible participants. Participants were approached at a convenient time for a face-to-face interview. A convenience sampling strategy was used. Participants with CKD grade 4 and 5 were also recruited from nephrology clinics. CKD grade 4 and 5 were verified using the estimated glomerular filtration rate (eGFR) from the most recent creatinine value with the CKD-EPI creatinine equation [34]. Data collection took approximately 12–18 min to complete.

### 3.4. Measurements

#### 3.4.1. Demographic and Clinical Characteristics

Data included age, sex, marital status, education level, monthly income, employment status, CKD grade and treatment modalities, treatment duration, and primary cause of CKD. These variables have been reported to influence decision-making [10,22]. Participants were assigned to one of four mutually exclusive clinical categories based on disease and treatment status: CKD G4, CKD G5 without KRT, hemodialysis, or peritoneal dialysis. Since treatment modality is relevant only after KRT initiation, CKD grade and treatment modality were combined into a single categorical variable to represent clinical status across the kidney disease trajectory. This combined variable was then utilized in comparative and regression analyses.

#### 3.4.2. Shared Decision-Making

Shared decision-making was measured using the 9-item Shared Decision-Making Questionnaire (SDM-Q-9) [5]. Each item is rated on a 6-point Likert scale from 0 (“completely disagree”) to 5 (“completely agree”). The Arabic version has demonstrated validity and reliability [35]. The SDM-Q-9 has been used across multiple populations (e.g., chronic obstructive pulmonary disease and type 2 diabetes) and in kidney populations [12,18,23,24,36]. Participants were instructed to complete the SDM-Q-9 based on the consultation or series of consultations in which treatment options were discussed and decisions regarding dialysis modality or future KRT were made. For participants already receiving dialysis, responses reflected their recollection of the modality selection process rather than their current treatment experience.

#### 3.4.3. Perception of Health Status and Comorbidities

Perceived health status was evaluated using the first self-rated health item adapted from the Arabic version of the Kidney Disease Quality of Life (KDQOL) questionnaire, with responses ranging from poor to excellent. The KDQOL questionnaire is a valid and reliable instrument that has been widely used in clinical research to assess individuals’ overall perception of health status. Comorbidity burden was assessed using the Davies Comorbidity Index [37]. Comorbidities were extracted from hospital records, coded as present/not present, and summed to create risk categories: 0 = low risk (no comorbidity); 1 = moderate risk (1–2 comorbidities); and 2 = high risk (3–7 comorbidities).

#### 3.4.4. Education and Support

Two dichotomous items assessed attendance at a kidney treatment options education class and availability of support from an informal caregiver. In this study, informal caregiver support was defined as a family member, partner, or friend who offers unpaid help with health-related decisions or daily care.

### 3.5. Data Analysis

Data were analyzed using the IBM Corp., Armonk, NY, USA, IBM Statistical Package for Social Sciences (SPSS), Version 26.0. No missing data were identified in any variables, as questionnaires were completed during face-to-face interviews and reviewed for completion at the time of data collection. Assessment of skewness, kurtosis, histograms, and Shapiro–Wilk tests indicated no substantial deviations from normality for continuous variables. Prior to regression analysis, assumptions were evaluated. Normality of residuals was assessed using histograms and normal probability (P–P) plots. Multicollinearity was examined using tolerance and variance inflation factor (VIF), independence of errors using the Durbin–Watson statistic, influential observations using standardized residuals, studentized deleted residuals, Cook’s distance, and leverage values, and homoscedasticity through visual inspection of standardized residual plots. Descriptive statistics (frequencies, percentages, means, and standard deviations) were calculated. Independent-samples *t*-tests and one-way ANOVA were used as appropriate to examine differences in SDM perceptions across participant characteristics. Levene’s test assessed homogeneity of variance. Bivariate Pearson correlations examined associations between SDM and selected variables. Stepwise multiple regression was used exploratively to identify variables independently associated with SDM, using entry criteria of *p* ≤ 0.05 and removal criteria of *p* ≥ 0.10. Multiple correlation (R) described the relationship between observed and predicted SDM scores. R^2^ represented the proportion of variance explained by predictors. Standardized beta coefficients (*β*) indicated the relative importance of predictors. The stepwise approach was used exploratively to identify variables independently associated with SDM; however, findings should be interpreted cautiously due to limitations associated with data-driven model selection. The primary objective of the regression analysis was exploratory, aiming at identifying variables independently associated with SDM rather than testing a pre-specified theoretical model. Therefore, stepwise multiple linear regression was used to identify predictors of SDM. This approach was considered appropriate given the limited evidence on SDM predictors in adults with advanced CKD in Saudi Arabia. However, as a data-driven method, the findings should be interpreted as exploratory and confirmed in future theory-driven studies.

### 3.6. Ethical Considerations

Ethical approval for this study was obtained from the Ministry of Health Institutional Review Board of the Riyadh Second Health Cluster for King Fahad Medical City (IRB no. 24-352E; 3 July 2024), and from the Institutional Review Board of King Khalid University Hospital, affiliated with King Saud University (IRB no. KSU-HE-23-742, 25 July 2024). Participants received written information describing study aims, procedures, risks, and benefits, and provided informed consent. Data were collected during hemodialysis, after hemodialysis, or in clinic settings, depending on participant preference. No identifying information was recorded. Participation was voluntary, and participants could withdraw at any time without consequences.

## 4. Results

### 4.1. Demographic and Clinical Characteristics of the Participants

A total of 294 questionnaires were analyzed. Table 1 summarizes the demographic and clinical characteristics of the participants. The mean age was 49.29 ± 15.470 years (range, 18–80). Most participants were male (58.8%), married (59.2%), and had Bachelor’s degrees (39.9%). In terms of socioeconomic status, a large proportion reported a monthly income below 5000 SR ≈ £1050 (53.1%), and many were unemployed (45.9%). Most were on hemodialysis (40.5%), followed by peritoneal dialysis (27.2%), while the pre-dialysis candidates were 32.3%; CKD grade 4 (17.7%) and CKD Grade 5 (14.6%), with a predicted treatment duration commonly 1–5 years (48.3%). Diabetes was the leading cause of CKD in 34.7% of our sample. The results also show that only 11.6% of participants reported having attended educational sessions on treatment options, whereas most reported receiving support from informal caregivers regarding health-related decisions or care (92.5%). This finding suggests limited access to structured pre-dialysis education within the participating settings. Overall, more than one-third of participants rated their health as fair (36.4%), while only a few reported excellent health (6.8%).

### 4.2. Perceptions of Shared Decision-Making

Participants’ perceptions of SDM were assessed. As shown in Table 2, the highest mean item scores were observed for “My doctor made clear that a decision needs to be made” (4.65 ± 1.861) and “My doctor helped me understand all the information” (4.60 ± 1.682). The lowest mean item scores were observed for “My doctor and I thoroughly weighed the different treatment options” (2.99 ± 2.164) and “My doctor and I selected a treatment option together” (3.00 ± 2.175). The overall mean SDM-Q-9 item score was 3.88 ± 1.476, corresponding to a standardized mean score of 64.7%.

### 4.3. Differences in Participant Decision-Making According to Sample Characteristics

The analysis of participants’ perceptions of SDM related to dialysis modality selection across demographic and clinical characteristics (Table 3) indicated that most demographic and clinical variables were not significantly associated with differences in perceptions. Specifically, no significant differences were observed for sex (*t* = 0.487; *p* = 0.627), age (*F* = 1.501; *p* = 0.177), education level (*F* = 1.995; *p* = 0.079), income status (*F* = 1.114; *p* = 0.350), CKD grades and treatment modalities (*F* = 0.437; *p* = 0.726) primary cause of CKD (*F* = 1.632; *p* = 0.138), or treatment onset time (*F* = 0.291; *p* = 0.884). In contrast, significant differences were found for Marital Status Groups (*F* = 6.286; *p* < 0.001) and employment status (*F* = 3.854; *p* = 0.022). Using Levene’s test, there was no difference between sexes regarding SDM (*F* = 0.487; *p* = 0.627).

### 4.4. Relationships Between Overall Shared Decision-Making Perceptions and Other Influencing Variables

Pearson correlation analyses were conducted to examine relationships between participants’ overall SDM perceptions and other influencing variables. Health perception was positively and significantly correlated with shared decision-making (*r* = 0.154 **; *p* = 0.008), indicating a low-strength relationship. Educational class attendance also showed a positive and significant association (*r* = 0.294 **; *p* < 0.001), representing a low-strength relationship. In contrast, informal caregiver support among adults with advanced CKD (*r* = 0.06; *p* = 0.308) and comorbidity status (*r* = −0.041; *p* > 0.05) were not significantly related to shared decision-making, suggesting negligible associations.

### 4.5. Predictors of SDM Among Adults with Advanced CKD

A multiple linear regression analysis was conducted to examine the extent to which attendance at a kidney treatment options education class and perception of health predicted shared decision-making perception. In Step 1, attendance at a kidney treatment options education class was entered. In Step 2, perception of health was added.

The overall model containing both predictors was statistically significant, *F*(2, 291) = 16.60, *p* < 0.001, and accounted for 10.2% of the variance in shared decision-making perception (*R*^2^ = 0.102, adjusted *R*^2^ = 0.096). The first model, which included only attendance at a kidney treatment options education class, was also significant, *F*(1, 292) = 27.67, *p* < 0.001, and explained 8.7% of the variance (*R*^2^ = 0.087, adjusted *R*^2^ = 0.083). The addition of perception of health in Step 2 produced a modest increase in explained variance (Δ*R*^2^ = 0.015). The standard error of the estimate decreased slightly from 0.474 in Model 1 to 0.470 in Model 2. The Durbin–Watson statistic was 1.955, indicating that the assumption of independent residuals was adequately met.

Collectively, these findings indicate that attendance at a kidney treatment options education class and perception of health together explain a small but statistically significant portion of the variance in shared decision-making perception.

Table 4 shows stepwise multiple regression results. It presents independent variables in the equation, that is, those factors that were found to be predictive of participants’ shared decision-making (*p* < 0.05). The other factors were not considered in the equation and were not found to be predictive of shared decision-making (*p* > 0.05), and these have high collinearity statistics results ranging from 0.54 to 0.97.

The multiple regression analysis showed that attendance at a kidney treatment options education class and perceived health were independently associated with SDM perception.

The regression model summary is presented in Table 5. In Model 1, attendance at a kidney treatment options education class explained 8.7% of the variance in SDM perception (*R*^2^ = 0.087). Adding perceived health in Model 2 increased the explained variance to 10.2% (*R*^2^ = 0.102; adjusted *R*^2^ = 0.096), representing a modest increase of 1.5 percentage points. The R indicates the direction of the correlation of the observed and predicted values of the dependent variable. Given this, model two (*R* = 0.320 correlation; *R*^2^ = 0.102) is the best model to represent the predictive factors of shared decision-making.

Based on the standardized coefficients in Table 4, A multiple regression was performed to evaluate the unique contributions of attendance at a kidney treatment options education class and perception of health to shared decision-making perception. In Model 1, attendance at a kidney treatment options education class significantly predicted shared decision-making perception, *B* = 0.454, *SE* = 0.086, *β* = 0.282, *t*(292) = 5.260, *p* < 0.001. Individuals who attended the class scored, on average, 0.454 units higher on the shared decision-making perception measure than those who did not attend.

In Model 2, both predictors were entered simultaneously. Attendance at a kidney treatment options education class remained a significant positive predictor, *B* = 0.436, *SE* = 0.086, *β* = 0.282, *t*(291) = 5.055, *p* < 0.001, after controlling for perception of health. Perception of health also emerged as a significant positive predictor, *B* = 0.056, *SE* = 0.025, *β* = 0.127, *t*(291) = 2.268, *p* = 0.024. For each one-unit increase in perception of health, shared decision-making perception increased by 0.06 units, holding education-class attendance constant. The standardized coefficients indicated that attendance at the education class (*β* = 0.282) exerted a stronger unique effect than perception of health (*β* = 0.127).

## 5. Discussion

In this study, we examined involvement in SDM among adults with advanced CKD and those receiving dialysis in Saudi Arabia in relation to dialysis modality decisions. Participants reported moderate to high levels of perceived involvement in SDM. These findings require cautious interpretation due to variability in the timing of decision-making across the sample. Specifically, participants undergoing long-term dialysis may have recalled consultations from much earlier periods compared to those with CKD G4 or G5. Greater involvement was associated with perceived health and exposure to educational interventions. These findings provide preliminary insights into how SDM is enacted within the Saudi kidney care context and contribute to the growing body of evidence, highlighting gaps between policy recommendations for person-centered kidney care and the lived experiences of adults and their involvement in making decisions about their treatment.

Overall, the findings suggest that while elements of SDM are present in clinical encounters, involvement may remain focused more on information exchange than on collaborative deliberation. This distinction is important because SDM extends beyond the provision of information and requires active engagement in discussing options, considering preferences, and reaching decisions collaboratively [38,39]. In the current study, participants generally perceived that healthcare professionals explained treatment information clearly and clarified that a decision was required. These findings suggest that aspects of “choice talk” and “option talk,” as described in Elwyn et al.’s [38] three-talk model of SDM, were relatively addressed. Few participants, however, reported being able to thoroughly weigh treatment options or jointly select a kidney treatment modality with healthcare professionals, suggesting that opportunities for collaborative deliberation were limited. This finding aligns with previous nephrology research indicating that clinical encounters often prioritize information provision over interactive decision-making processes [40]. Adults with advanced CKD may therefore feel informed about treatment options without being fully engaged in deliberate discussions regarding what matters most to them in decision-making.

The interpretation of these findings requires consideration of the healthcare and cultural context in which decisions are made. In Saudi Arabia, clinical decision-making remains largely physician-led, which may influence patient participation in SDM processes, and the decision-making process is often constrained by organizational and regulatory structures [41]. While nurses have an essential role in patient education and support, opportunities for independent or nurse-led SDM processes remain limited compared with some Western healthcare systems. Consequently, SDM may be shaped largely by medical practitioners’ communication styles and the extent to which adults are invited to participate actively in treatment discussions. Cultural factors, including trust in medical practitioners, respect for medical authority, religious beliefs, and family involvement in healthcare decisions, may further influence how adults with advanced CKD experience and report involvement in decision-making [28,29]. These contextual influences warrant further investigation within Saudi nephrology settings [42]. The Saudi healthcare context significantly shapes the implementation of SDM in nephrology care. Despite substantial progress toward person-centered care under the Saudi Vision 2030 healthcare transformation, clinical decision-making in Saudi Arabia remains predominantly clinician-led, with physicians holding primary responsibility for treatment recommendations [29]. Moreover, family members often play a central role in healthcare decisions, especially for patients with advanced or complex illnesses. Although family involvement offers valuable emotional and practical support, it can also affect how patients articulate their treatment preferences during clinical consultations. Additionally, cultural values that emphasize respect for healthcare professionals and trust in clinical expertise may prompt some adults to defer treatment decisions to clinicians rather than engage actively in deliberation [43]. These contextual factors do not preclude SDM but underscore the need for culturally sensitive strategies that promote patient participation while recognizing the supportive role of families in treatment decisions. One possible explanation is that clinical decision-making in Saudi Arabia has traditionally been physician-led, which may limit opportunities for patient participation in SDM. Previous studies indicate that cultural expectations, family involvement, and established clinician–patient relationships shape patients’ preferences for involvement in healthcare decisions, especially in contexts where family-centered care and clinician authority are prioritized [44,45,46].

Decision-making in advanced CKD extends beyond selecting a dialysis modality and may also include the option of conservative kidney management. Such decisions are complex and involve trade-offs related to quality of life, symptom burden, and survival [39]. However, conservative management pathways remain inconsistently implemented in many Middle Eastern healthcare systems, including Saudi Arabia [47]. Financial limitations, institutional barriers, and cultural or religious considerations may restrict discussion of non-dialysis pathways [47]. In settings where treatment decisions are framed primarily around clinical recommendations, adults may perceive limited opportunity to consider alternative options. Meaningful SDM, therefore, requires that all reasonable treatment options, including conservative management, are explicitly discussed and aligned with individuals’ values and preferences [13]. Although this study focused on shared decision-making rather than the determinants of dialysis modality selection, treatment choices are influenced by multiple clinical and personal factors. Patients may consider perceived treatment burden, infection risk, home- versus center-based treatment, dialysis-related complications, and the impact of treatment on daily life, while healthcare professionals also consider clinical suitability when making recommendations [3,8,15,20]. Health literacy, family involvement, and sociocultural factors further shape dialysis decisions. Individuals with a greater understanding of their treatment options are generally better able to participate in decision-making, whereas family support, cultural values, religious beliefs, and trust in healthcare professionals may influence how treatment options are considered [12,22,43,48]. Therefore, dialysis modality selection should be viewed as a multifaceted process in which shared decision-making integrates clinical evidence with patients’ preferences, values, and life circumstances [3,20].

Participants reported relatively high perceptions of involvement compared with previous studies [22,24,49], although these findings should be interpreted with caution. SDM was assessed using a self-report measure, which may overestimate actual involvement in decision-making processes. Previous research has shown that adults’ perceptions of involvement do not always reflect the extent to which decisions are genuinely shared, particularly within clinician-led consultations [26]. Participants may perceive that they received adequate information while still having limited opportunity to question recommendations, compare alternatives, or express preferences [42,50]. Differences in cultural norms, healthcare systems, and study designs may also contribute to variation across studies.

Several factors were associated with SDM in this study, including educational class attendance and perceived health. Only a small proportion of participants reported attending formal education sessions regarding kidney treatment options, which is consistent with previous evidence demonstrating limited participation in pre-dialysis education programs [18]. This may reflect variation in access to structured education across institutions and regions within Saudi Arabia [51]. In this study, we found that attendance at educational sessions was associated with higher SDM scores, although this relationship should be interpreted cautiously because individuals who attend educational programs may already be more motivated or better prepared to participate in treatment decisions. Furthermore, evidence suggests that education alone may be insufficient to support involvement in SDM meaningfully. More interactive approaches that encourage discussion, deliberation, and patient participation may better support engagement in treatment decisions [3,39,52].

Perceived health status was also associated with SDM, suggesting that engagement in decision-making is shaped by both individual and social factors. Adults who perceive their health more positively may be better able to participate in complex treatment discussions [18].

Family support is particularly relevant within the Saudi cultural context, where family involvement in healthcare decisions is common and often expected [53]. However, it was not significantly associated with SDM in this study. This may suggest that the presence of family support does not necessarily influence the extent to which patients are involved in shared decision-making. While family support may help patients better understand and participate in care, SDM approaches should continue to maintain the patient’s central role in treatment decisions [53].

Although the regression model explained a modest proportion of variance in SDM, this is consistent with the multidimensional nature of healthcare decision-making. SDM is influenced not only by the person’s characteristics but also by clinician communication, healthcare structures, and broader cultural and organizational factors. These findings suggest that strengthening SDM in kidney care requires broader organizational and relational approaches rather than education-focused strategies alone.

### Limitations

Several limitations should be considered when interpreting these findings. First, the cross-sectional design does not permit causal inference between identified predictors and SDM involvement. Therefore, factors such as educational attendance or timing of information provision should be interpreted as associated with SDM rather than causally influencing it. Second, SDM was assessed using a self-report instrument, which captures adults’ perceptions of clinician behaviors rather than the reciprocal and interactive nature of SDM itself. The SDM-Q-9 specifically evaluates patients’ perceptions of physician behaviors during shared decision-making consultations. Consequently, the present study cannot determine the independent contributions of nephrology nurses or other members of the multidisciplinary kidney-care team. Consequently, findings may reflect perceived involvement rather than actual shared deliberation and may be subject to recall, response, and social desirability biases [22]. Furthermore, the timing of the decision-making encounter differed among participants. Adults with CKD G4 or G5 typically reported recent treatment planning discussions, while participants already undergoing dialysis often recalled consultations from months or years prior to questionnaire administration. As the interval between dialysis modality selection and survey completion was not documented, it was not possible to assess the impact of the recall period or to stratify analyses by decision timing. Therefore, retrospective responses may have been affected by recall bias or by participants’ subsequent treatment experiences. Participants were recruited via convenience sampling from two hospitals in Saudi Arabia, which may have introduced selection bias. Individuals who agreed to participate may differ from individuals who declined or from adults receiving care in other healthcare settings. Therefore, the findings may not be fully representative of all adults with advanced kidney disease in Saudi Arabia and should be interpreted with caution. Additionally, the study was conducted in only two centers, which may limit transferability to healthcare systems with different organizational structures, cultural norms, and models of patient–provider interaction. Contextual factors such as physician-led decision-making traditions and variability in access to structured SDM support may also have influenced the observed level of SDM. Recruitment logs detailing the numbers of individuals assessed for eligibility, approached, declining participation, or excluded at each participating center were not maintained. As a result, it was not possible to calculate center-specific response rates or compare participants with non-participants, because data on individuals who declined to participate were not collected. This limitation restricts the ability to assess potential selection bias. Furthermore, the regression model accounted for 10.2% of the variance in SDM, suggesting that additional unmeasured factors may influence patient involvement. Potentially important factors, including health literacy, patient–physician communication, decisional conflict, patient preferences, and organizational factors, were outside the scope of this study and were not included in the regression analysis. Future research should incorporate these factors to develop more comprehensive models of SDM among adults with advanced CKD.

## 6. Implications for Practice and Research

The findings have important implications for clinical practice and future research. Attendance at a kidney treatment options education class and perceived health status were independently associated with SDM perception in the final regression model. However, because of the cross-sectional design, these associations should not be interpreted as evidence that education or perceived health status directly enhances SDM. Individuals who attended educational sessions may have differed from non-attendees in motivation, health literacy, prior preparation, or other unmeasured characteristics. Notably, the association between educational class attendance and higher SDM scores suggests that structured education warrants further investigation. However, due to the cross-sectional design of this study, these associations should not be interpreted as evidence that education or earlier decision support directly enhances SDM. Individuals who attended educational sessions may have differed from non-attendees in motivation, health literacy, prior preparation, or other unmeasured characteristics.

From a practice perspective, the findings may support consideration of timely and structured opportunities for patients to receive information about available treatment options and to discuss their preferences and values. However, the effectiveness of specific educational or decision-support interventions cannot be established from the present study. Evidence from previous intervention research suggests that structured decision-support approaches, including patient decision aids and decision coaching, may facilitate patient involvement in kidney treatment decisions [13,18]. These approaches may be considered within multidisciplinary kidney-care practice, while their effectiveness and implementation in Saudi nephrology settings require further evaluation.

Future research should use longitudinal and intervention-based designs to determine whether the timing, content, and delivery of education or decision support influence SDM over time. Studies should also examine the roles of nephrologists, nephrology nurses, dietitians, and other members of the multidisciplinary kidney-care team in supporting SDM. Additional research is needed to investigate psychosocial and system-level factors, including health literacy, decisional conflict, clinician communication styles, patient preferences, and institutional constraints. More objective approaches to assessing SDM, such as consultation observation or audio/video analysis, may also help determine whether interventions improve actual collaborative deliberation rather than only patients’ perceptions of SDM.

## 7. Conclusions

This study provides important insight into SDM in dialysis modality selection among adults with advanced CKD in Saudi Arabia, demonstrating moderate-to-high patient-reported involvement, although collaborative deliberation appeared inconsistently implemented. These findings suggest increasing attention to person-centered care and that SDM may remain inconsistently implemented and focused more on information exchange than collaborative deliberation.

The identification of factors independently associated with SDM, particularly attendance at a kidney treatment options education class and perceived health status, provides insight into factors that may be related to patient involvement in treatment decisions. These findings suggest that strengthening SDM in kidney care requires approaches that extend beyond information provision alone and support meaningful patient engagement throughout the decision-making process. Nurses and other healthcare professionals have an important role in facilitating the involvement of adults with CKD through communication, education, and multidisciplinary collaboration. Future longitudinal and multi-center studies are needed to better understand how SDM evolves across the CKD trajectory and how structured SDM approaches can be effectively implemented within diverse cultural and healthcare contexts. Although we assessed patients’ perceptions of physician involvement in SDM in this study, the literature indicates that multidisciplinary kidney care teams may enhance SDM by providing structured education, decision support, and patient-centered communication. These implementation strategies should be evaluated in future research.

## Figures and Tables

**Table 1:** Demographic and clinical characteristics (*N* = 294).

Characteristics	Number (*n*)	Percentage (%)
Age, *Mean*, *SD*	49.29 ± 15.470	
Sex		
Male	173	58.8%
Female	121	41.2%
Marital Status		
Single	72	24.4%
Married	174	59.2%
Divorced	24	8.2%
Widowed	24	8.2%
Education Level		
No Formal Education	21	7.1%
Primary Education	28	9.5%
Intermediate Education	35	11.9%
Secondary Education	73	24.8%
Undergraduate (Bachelor’s Degree)	117	39.9%
Postgraduate Education	20	6.8%
Income Status		
Less than 5000	156	53.1%
5000–10,000	51	17.3%
10,001–20,000	67	22.8%
20,001–30,000	11	3.7%
More than 30,000	9	3.1%
Employment Status		
Employed	86	29.3%
Unemployed	135	45.9%
Retired	73	24.8%
Clinical Disease and Treatment Status		
CKD G4	52	17.7%
CKD G5 without KRT	43	14.6%
Hemodialysis	119	40.5%
Peritoneal Dialysis	80	27.2%
Time Since Dialysis Initiation or First Nephrology Clinic Visit		
Less than 6 Months	43	14.6%
6 Months–1 Year	35	11.9%
1–5 Years	142	48.3%
6–10 Years	40	13.6%
More than 10 Years	34	11.6%
Primary Cause of Advanced CKD		
Diabetes	102	34.7%
Hypertension	58	19.7%
Obstructive Nephropathy	1	0.3%
Polycystic Kidney Disease	7	2.4%
Chronic Glomerulonephritis	3	1.0%
Unknown Etiology	40	13.6%
Others	83	28.3%
Comorbidity		
Grade 0 (No Comorbidities)	87	29.6%
Grade 1 (1–2 Comorbidities)	206	70.1%
Grade 2 (3–7 Comorbidities)	1	0.3%
Educational Class Attendance		
Yes	34	11.6%
No	260	88.4%
Informal Caregiver Support		
Yes	272	92.5%
No	22	7.5%
Perception of Health		
Excellent	20	6.8%
Very Good	33	11.2%
Good	82	27.9%
Fair	107	36.4%
Poor	52	17.7%

**Table 2:** Participant shared decision-making (*N* = 294).

The 9-Item Shared Decision-Making Questionnaire	*Mean*	*SD*	%
My doctor made clear that a decision needs to be made	4.65	1.861	77.50
My doctor wanted to know exactly how I want to be involved in making the decision	4.22	1.968	70.33
My doctor told me that there are different options for treating my medical condition	4.39	2.024	73.17
My doctor precisely explained the advantages and disadvantages of the treatment options	3.39	2.168	56.50
My doctor helped me understand all the information	4.60	1.682	76.67
My doctor asked which treatment option I prefer.	3.34	2.273	55.67
My doctor and I thoroughly weighed the different treatment options.	2.99	2.164	49.83
My doctor and I selected a treatment option together.	3.00	2.175	50.00
My doctor and I reached an agreement on how to proceed.	4.32	2.037	72.00
Mean of SDM-Q-9 item score (ranged 0–5)	3.88	1.476	
Sum of SDM-Q-9 item range	0–45		

**Table 3:** Differences in decision-making based on sample characteristics.

Variables	Category	Mean Score	*SD*	Test Statistic	*df*	*p*
Sex	Male	1.43	0.493	*t* = 0.487	(292)	0.627
	Female	1.40				
Age	Age Groups	49.29	15.470	*F* = 1.501	(6, 287)	0.177
Marital Status	Marital Status Groups	2.00	0.809	*F* = 6.286	(3, 290)	<0.001 **
Educational Level	Educational Level Groups	3.44	1.275	*F* = 1.995	(5, 288)	0.079
Income Status	Income Groups	1.86	1.081	*F* = 1.114	(4, 289)	0.350
Employment Status	Employment Groups	1.96	0.735	*F* = 3.854	(2, 291)	0.022 *
CKD Grade and Treatment Modality	CKD/Treatment Groups	2.77	1.038	*F* = 0.437	(3, 290)	0.726
Time Since Dialysis Initiation or First Nephrology Clinic Visit	Time Groups	2.96	1.142	*F* = 0.291	(4, 289)	0.884
Primary Cause of AKD	Cause Groups	3.69	2.634	*F* = 1.632	(6, 287)	0.138

Note: SDM = shared decision-making. An independent-samples *t*-test was used for dichotomous variables and one-way ANOVA for variables with more than two categories. * *p* < 0.05; ** *p* < 0.001.

**Table 4:** Predictors of SDM identified by stepwise multiple linear regression analysis.

Variables	*B*	*SE*	*β*	*t*	*p*
Model 1					
(Constant)	0.915	0.100		9.128	<0.001
Have you attended a kidney treatment options education class?	0.454	0.086	0.294	5.260	<0.001
Model 2					
(Constant)	0.741	0.126		5.893	<0.001
Have you attended a kidney treatment options education class?	0.436	0.086	0.282	5.055	<0.001
Perception of Health	0.056	0.025	0.127	2.268	0.024

Note: Shared decision-making was the dependent variable; *β* = standardized regression coefficient; *B* is the unstandardized coefficient; *SE* is the standard error.

**Table 5:** Model summary of regression analysis of level of shared decision-making.

Model	*R*	*R* ^2^	Adjusted *R*^2^	Std. Error
1	0.294 ^a^	0.087	0.083	0.474
2	0.320 ^b^	0.102	0.096	0.470

Dependent Variable: Shared-Decision-Making Perception. Independent Variables: “ ^a^ [Have you attended a kidney treatment options education class]; ^b^ [Have you attended a kidney treatment options education class, Perception of Health].”

## Data Availability

The dataset is available on request from the authors.

## Supplementary Materials

The following supporting information can be downloaded at https://www.mdpi.com/article/10.3390/healthcare14162610/s1. Supplementary File S1: STROBE Statement—Checklist of items that should be included in reports of cross-sectional studies.

## Abbreviations

The following abbreviations are used in this manuscript:

CKD	Chronic kidney disease
SDM	Shared decision-making
SDM-Q-9	9-item Shared Decision-Making Questionnaire

## References

[1] Amoah W.W., Ihudiebube-Splendor C., Dzramado V.L. (2025). Impact of chronic kidney disease on health-related quality of life in adults: A systematic review and meta-analysis protocol. Front. Nephrol..

[2] Kovesdy C.P. (2022). Epidemiology of chronic kidney disease: An update 2022. Kidney Int. Suppl..

[3] Yu X., Nakayama M., Wu M.S., Kim Y.L., Mushahar L., Szeto C.C., Schatell D., Finkelstein F.O., Quinn R.R., Duddington M. (2022). Shared decision-making for a dialysis modality. Kidney Int. Rep..

[4] Elwyn G., Gulbrandsen P., Leavitt H., Abukmail E., Clayman M.L., Edwards A., Finderup J., Fisher A., Grande S.W., Hahlweg P. (2025). Shared decision-making. A primer for clinicians. J. Gen. Intern. Med..

[5] Kriston L., Scholl I., Hölzel L., Simon D., Loh A., Härter M. (2010). The 9-item shared decision making questionnaire (SDM-Q-9). Development and psychometric properties in a primary care sample. Patient Educ. Couns..

[6] Bekker H.L., Finderup J., Mooney A., Søndergaard H., Winterbottom A.E., Elwyn G., Joseph-Williams N., Edwards A. (2025). Shared Decision Making Interventions for Kidney Failure Treatment and Management. Oxford Textbook of Shared Decision Making in Healthcare.

[7] de-la-Motte P., Jensen V.B.J., Bergum M.H., Mose F.H., Khatir D.S., Finderup J. (2025). Shared decision making and dialysis choice: An observational longitudinal cohort study. BMC Nephrol..

[8] Campbell-Montalvo R., Jia H., Shukla A.M. (2022). Supporting shared decision-making and home dialysis in end-stage kidney disease. Int. J. Nephrol. Renov. Dis..

[9] Easom A.M., Shukla A.M., Rotaru D., Ounpraseuth S., Shah S.V., Arthur J.M., Singh M. (2020). Home run-results of a chronic kidney disease Telemedicine Patient Education Study. Clin. Kidney J..

[10] Engels N., De Graav G., Van Der Nat P., Van Den Dorpel M., Bos W.J., Stiggelbout A.M. (2020). Shared decision-making in advanced kidney disease: A scoping review protocol. BMJ Open.

[11] Cassidy B.P., Getchell L.E., Harwood L., Hemmett J., Moist L.M. (2018). Barriers to education and shared decision making in the chronic kidney disease population: A narrative review. Can. J. Kidney Health Dis..

[12] Finderup J., Jensen J.D., Lomborg K. (2021). Shared decision-making in dialysis choice has potential to improve self-management in people with kidney disease: A qualitative follow-up study. J. Adv. Nurs..

[13] Brown L., Gardner G., Bonner A. (2019). A randomized controlled trial testing a decision support intervention for older patients with advanced kidney disease. J. Adv. Nurs..

[14] Cherry K., Stacey D., Finderup J. (2024). 042 Decision support interventions for dialysis choice: A structured process of shared decision making for a patient with chronic kidney disease. A case study. Abstract for Poster Submission.

[15] Chan C.T., Blankestijn P.J., Dember L.M., Gallieni M., Harris D.C.H., Lok C.E., Mehrotra R., Stevens P.E., Wang A.Y.M., Cheung M. (2019). Dialysis initiation, modality choice, access, and prescription: Conclusions from a Kidney Disease: Improving Global Outcomes (KDIGO) Controversies Conference. Kidney Int..

[16] Hassan R., Akbari A., Brown P.A., Hiremath S., Brimble K.S., Molnar A.O. (2019). Risk factors for unplanned dialysis initiation: A systematic review of the literature. Can. J. Kidney Health Dis..

[17] Shimizu Y., Nakata J., Yanagisawa N., Shirotani Y., Fukuzaki H., Nohara N., Suzuki Y. (2020). Emergent initiation of dialysis is related to an increase in both mortality and medical costs. Sci. Rep..

[18] Frazier R., Levine S., Porteny T., Tighiouart H., Wong J.B., Isakova T., Koch-Weser S., Gordon E.J., Weiner D.E., Ladin K. (2022). Shared decision making among older adults with advanced CKD. Am. J. Kidney Dis..

[19] Truglio-Londrigan M., Slyer J.T. (2018). Shared decision-making for nursing practice: An integrative review. Open Nurs. J..

[20] Amir N., McCarthy H.J., Tong A. (2021). A working partnership: A review of shared decision-making in nephrology. Nephrology.

[21] Chou C.Y., Hsieh Y.L., Lai J.W. (2025). Who decides on peritoneal dialysis treatment? A decision analysis for patients with kidney failure. J. Health Psychol..

[22] Finderup J., Lomborg K., Jensen J.D., Stacey D. (2020). Choice of dialysis modality: Patients’ experiences and quality of decision after shared decision-making. BMC Nephrol..

[23] Ghodsian S., Ghafourifard M., Ghahramanian A. (2021). Comparison of shared decision making in patients undergoing hemodialysis and peritoneal dialysis for choosing a dialysis modality. BMC Nephrol..

[24] Schellartz I., Ohnhaeuser T., Mettang T., Scholten N. (2021). Information about different treatment options and shared decision making in dialysis care—A retrospective survey among hemodialysis patients. BMC Health Serv. Res..

[25] Bakker J., Huntink E.M., Peters L.J., Brugman I.M., Ubbink D.T., Schoonhoven L. (2025). Factors influencing shared decision-making on hospital wards as perceived by healthcare professionals: A qualitative study. Appl. Nurs. Res..

[26] Berger-Höger B., Liethmann K., Mühlhauser I., Haastert B., Steckelberg A. (2019). Nurse-led coaching of shared decision-making for women with ductal carcinoma in situ in breast care centers: A cluster randomized controlled trial. Int. J. Nurs. Stud..

[27] Fisher K.A., Tan A.S.L., Matlock D.D., Saver B., Mazor K.M., Pieterse A.H. (2018). Keeping the patient in the center: Common challenges in the practice of shared decision making. Patient Educ. Couns..

[28] AlHaqwi A.I., AlDrees T.M., AlRumayyan A., AlFarhan A.I., Alotaibi S.S., AlKhashan H.I., Badri M. (2015). Shared clinical decision making. A Saudi Arabian perspective. Saudi Med. J..

[29] Aljaffary A., Alsheddi F., Alzahrani R., Alamoudi S., Aljuwair M., Alrawiai S., Aljabri D., Althumairi A., Hariri B., Alumran A. (2022). Shared decision-making: A cross-sectional study assessing patients awareness and preferences in Saudi Arabia. Patient Prefer. Adherence.

[30] Alrawiai S., Aljaffary A., Al-Rayes S., Alumran A., Alhuseini M., Hariri B. (2020). The Option scale: Measuring patients’ perceptions of shared decision-making in the Kingdom of Saudi Arabia. J. Multidiscip. Healthc..

[31] Amin H.S., Arafa M.A., Farhat K.H., Rabah D.M., Altaweel A.A., Alhammad A.H. (2020). Does shared decision making increase prostate screening uptake in countries with a low prevalence of prostate cancer?. Afr. Health Sci..

[32] Alkhaibari A.S., Altabbaa A.M., Albeladi D.A., Alharbie M.K.N., Aljohani R.M., Almuzaini A.S., Alkhaibari S.S., Fallatah S.A., Aljawi B.S., Altabbaa K.M. (2024). Determinants of shared decision-making awareness among hemodialysis nurses: The impact of professionalism, empathy, and clinical decision-making. Rev. Diabet. Stud..

[33] Harrell F.E. (2015). Regression Modeling Strategies.

[34] Johnson D.W., Jones G.R.D., Mathew T.H., Ludlow M.J., Doogue M.P., Jose M.D., Langham R.G., Lawton P.D., McTaggart S.J., Peake M.J. (2012). Chronic kidney disease and automatic reporting of estimated glomerular filtration rate: New developments and revised recommendations. Med. J. Aust..

[35] Alzubaidi H., Hussein A., Mc Namara K., Scholl I. (2019). Psychometric properties of the Arabic version of the 9-item Shared Decision-Making Questionnaire: The entire process from translation to validation. BMJ Open.

[36] Nørgaard B., Titlestad S.B., Marcussen M. (2022). Shared decision-making in general practice from a patient perspective. A cross-sectional survey. Scand. J. Prim. Health Care.

[37] Davies S.J., Phillips L., Naish P.F., Russell G.I. (2002). Quantifying comorbidity in peritoneal dialysis patients and its relationship to other predictors of survival. Nephrol. Dial. Transplant..

[38] Elwyn G., Durand M.A., Song J., Aarts J., Barr P.J., Berger Z., Cochran N., Frosch D., Galasiński D., Gulbrandsen P. (2017). A three-talk model for shared decision making: Multistage consultation process. BMJ.

[39] Stacey D., Lewis K.B., Smith M., Carley M., Volk R., Douglas E.E., Pacheco-Brousseau L., Finderup J., Gunderson J., Barry M.J. (2024). Decision aids for people facing health treatment or screening decisions. Cochrane Database Syst. Rev..

[40] Andersen-Hollekim T., Landstad B.J., Solbjør M., Kvangarsnes M., Hole T. (2021). Nephrologists’ experiences with patient participation when long-term dialysis is required. BMC Nephrol..

[41] Alenezi I.N. (2026). Workplace barriers to nursing autonomy in Saudi hospitals: Implications for management and policy under vision 2030. J. Nurs. Manag..

[42] Alfattani M.A., Bakudam W.A., Alharbi W.I. (2024). Factors influencing patient-centered care and shared decision-making in primary care settings in Saudi Arabia: A scoping review. Cureus.

[43] Cotta A., Kristiansen M. (2023). Enacting person-centred care: A multi-perspective study of practices in clinical encounters for people living with chronic kidney disease. BMC Nephrol..

[44] Park H., Jung T. (2024). The effects of equine-assisted learning on adolescents with Internet gaming disorder. Healthcare.

[45] Almutairi A.F., McCarthy A., Gardner G.E. (2014). Understanding cultural competence in a multicultural nursing workforce: Registered nurses’ experience in Saudi Arabia. J. Transcult. Nurs..

[46] Elwyn G., Frosch D., Thomson R., Joseph-Williams N., Lloyd A., Kinnersley P., Cording E., Tomson D., Dodd C., Rollnick S. (2012). Shared decision making: A model for clinical practice. J. Gen. Intern. Med..

[47] Koubar S.H., Hatab T., Razzak F.A., Helal I., Ali A., Shebani A., Kaysi S., Gunderman D., Al-Makki A., Davison S.N. (2025). Conservative kidney management in the Middle East and North Africa: Attitudes, practices, and implementation barriers. Kidney Int. Rep..

[48] Morton R.L., Sellars M. (2019). From patient-centered to person-centered care for kidney diseases. Clin. J. Am. Soc. Nephrol..

[49] Robinski M., Mau W., Wienke A., Girndt M. (2016). Shared decision-making in chronic kidney disease: A retrospection of recently initiated dialysis patients in Germany. Patient Educ. Couns..

[50] Alkhaibari R.A., Smith-Merry J., Forsyth R., Raymundo G.M. (2023). Patient-centered care in the Middle East and North African region: A systematic literature review. BMC Health Serv. Res..

[51] Al-Hazmi A.I., Alghamdi M.A., Alzahrani R.S., Alzahrani S.S., Alibrahim S.R., Alharbi A.A., Alghamdi M.A., Alzahrani F.A., Alghamdi T.S., Alharbi S.A. (2022). Barriers to pre-dialysis education in hemodialysis patients with end-stage renal disease in Kingdom of Saudi Arabia. Middle East J. Fam. Med..

[52] Faldu C.T., Knicely D.H. (2025). Advanced chronic kidney disease and patient education. Kidney Dial..

[53] Alfahmi M.Z. (2022). Patients’ preference approach to overcome the moral implications of family-centred decisions in Saudi medical settings. BMC Med. Ethics.

